# Proteome characterization of a human urothelial cell line resistant to the bladder carcinogen 4-aminobiphenyl

**DOI:** 10.1186/1477-5956-5-6

**Published:** 2007-05-03

**Authors:** Roberta Pastorelli, Federica Saletta, Donatella Carpi, Roberta Campagna, Carlo dell'Osta, Silvia Schiarea, Paolo Vineis, Luisa Airoldi, Giuseppe Matullo

**Affiliations:** 1 Department of Environmental Health Sciences, Istituto di Ricerche Farmacologiche Mario Negri, Milano, Italy; 2 Section of Life Sciences, Institute for Scientific Interchange Foundation, Torino, Italy; 3 Department of Epidemiology and Public Health, Imperial College London, London, UK; 4 Department of Genetics, University of Torino, Torino, Italy; 5 Department of Biomedical Sciences and Human Oncology, University of Torino, Torino, Italy

## Abstract

**Background:**

The aromatic amine 4-aminobiphenyl (4-ABP) is an environmental and occupational contaminant known to be a major etiological agent of human bladder cancer. 4-ABP metabolites are able to form DNA adducts that may induce mutations and initiate bladder carcinogenesis. Cells exposed to 4-ABP may develop resistance to the carcinogen. The aim of the present study was to detect and identify proteins whose expression is altered in the bladder carcinoma RT112 sub-lines selected for acquired resistance to 4-ABP, in order to disentangle the mechanisms.

**Results:**

Differential proteome analysis of cell lysates showed an overall perturbation in cell metabolism and energy pathways in the 4-ABP-resistant human urothelial clones, with over-expression of membrane trafficking proteins such as annexin 2. The resistant clones had altered expression of many proteins linked directly (*i.e*. lamin A/C, programmed cell death 6 interacting protein) or indirectly (*i.e*. 94 kDa glucose-regulated protein, fatty acid-binding protein) to decreased apoptosis, suggesting that resistance to 4-ABP might be associated with low apoptotic activity.

**Conclusion:**

Our data provide evidence that deregulation of apoptosis and membrane trafficking proteins might be strongly implicated in the selection of carcinogen resistant cells. Some of these proteins might have potential as biomarkers of resistance and cancer risk.

## Background

Resistance is a complex process, very likely the result of multiple and often overlapping routes that can be affected by a variety of host and acquired factors often not clearly defined. Proteomic techniques, that allow observation of changes in multiple proteins at once, might play a key role in understanding the development of cell resistance to drugs, helping clarify the mechanisms through which cells escape their effects [1-3].

Although the multifactorial aspect of resistance still needs to be disentangled, it is generally accepted that the selective pressure exerted by drugs, combined with cell heterogeneity, is the driving force for drug resistance. Specific carcinogens, that can form adducts to DNA, can even select carcinogen-resistant tumour cells [4] that may have sustained extensive DNA damage yet somehow escape death. The phenotypic and potential genotypic differences in the surviving cells may convey a selective survival advantage, which may disrupt cell death/growth homeostasis and predispose these cells to progression. Thus, as anticancer agents drive carcinogenesis by means of natural selection, environmental agents might act in the same manner. As an example, carcinogens from inhaled tobacco smoke not only induce mutations, but also set up a selection pressure that favours mutants resistant to the cytotoxic effects of smoking [5].

We have already observed that in bladder cells the DNA bulky adducts formed by the carcinogen 4-aminobiphenyl (4-ABP) were proportionate to the tumour grades [6], suggesting again that cancer development can lead to "tolerance" to DNA damage, through clonal cell selection. To date, there is no clear characterisation of this phenotype and the mechanisms by which cells develop resistance toward an environmental carcinogen are poorly understood. It is likely that a combination of several factors is involved, as in drug resistance. Thus a proteomic approach is of interest to compare protein profiles between carcinogen-resistant cells and their non-resistant counterparts.

In the present study we treated the human transitional bladder cell carcinoma line RT112 with 4-ABP to select for cells developing resistance to this compound. 4-ABP is an environmental and occupational contaminant that can interact with DNA to form bulky adducts through its metabolically activated electrophilic derivatives, and induces bladder carcinogenesis in humans [6].

We used a proteomic approach to examine the overall protein expression profile and characterize its alteration in sub-lines selected for resistance to 4-ABP, in order to gain insight into *(i) *the mechanisms involved in the cells' response to 4-ABP exposure, and *(ii) *the potential mechanisms by which resistance can arise.

## Results

### Resistant clones

**Figure 1:**
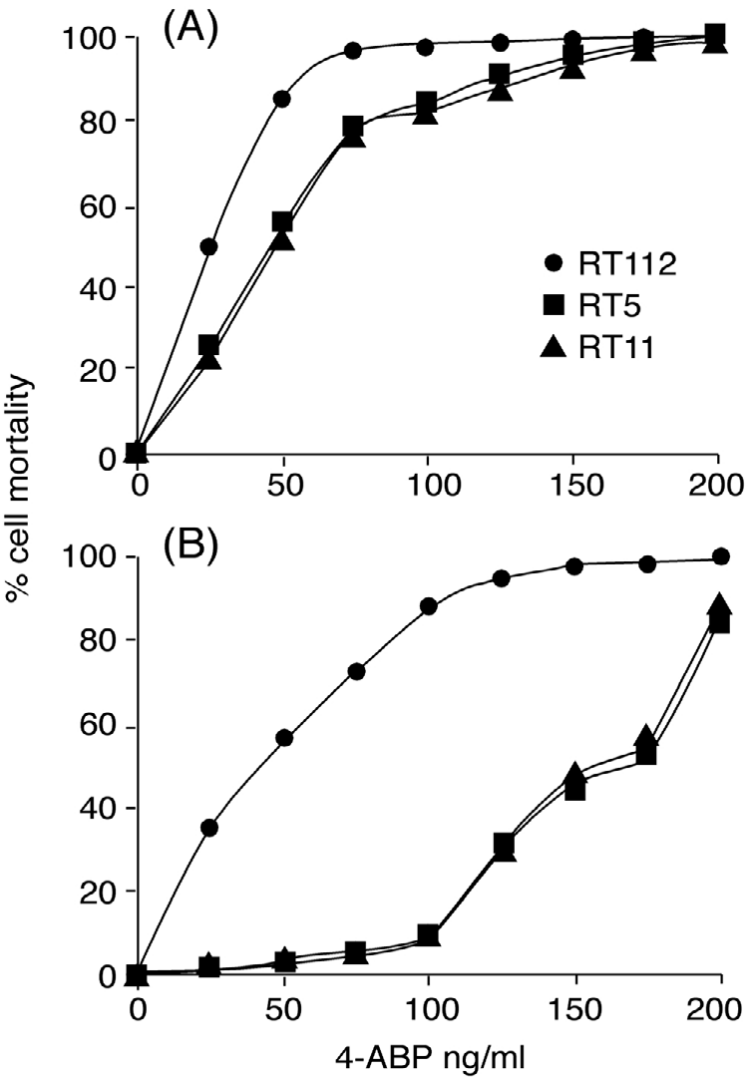
Cytotoxicity curves for human bladder cancer cell line RT112 and 4-ABP resistant clones RT5 and RT11 after the first 4-ABP treatment panel (A); after the second 4-ABP treatment, panel (B).

The first step was to establish derivatives of the RT112 cell line resistance to the carcinogen 4-ABP. We treated cells with a panel of carcinogen concentrations in order to obtain cytotoxicity curves. Details of selected 4-ABP resistant clones will be reported in a forthcoming publication (F. Saletta et al., submitted). Briefly, 4-ABP resistant cells were established by treatment with a carcinogen concentration that normally kills > 99% of RT112 cells (125 ng/mL). After treatment, viable single-cell clones were obtained by limiting dilution. The relative resistance of isolated clones was evaluated by treating cells with 4-ABP (75–175 ng/mL) and viability was measured by Trypan Blue. Thus, we developed two cell sublines, RT5 and RT11, showing a high degree of resistance (respectively 90% and 88% after first treatment of 125 ng/mL 4-ABP; 30% and 31% after second treatment of 125 ng/mL 4-ABP as shown in figure 1).

### Proteomic analysis

The expression patterns of proteins of the 4-ABP-resistant RT5 and RT11 clones were compared with the parental RT112 cells using 2-DE image analysis software.

**Figure 2:**
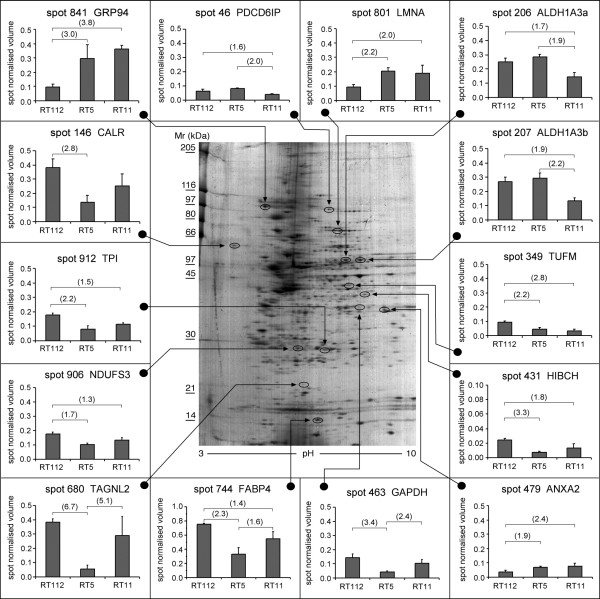
Colloidal CBB-stained 2-DE gel (Progenesis average gel image) of cell protein extract (300 μg) from human bladder cancer cell line RT112. The inserts show the expression patterns for the selected protein species whose abundance changed significantly (one-way ANOVA, Tukey Kramer HSD p < 0.05) in the human bladder cancer cell line RT112 and the 4-ABP resistant clones RT5 and RT11. Each bar represents the average spot abundance expressed as normalised volume ± SD. The vertical axis shows the spot normalised volume. Numbers in parentheses indicate protein expression difference (-fold). Spot numbers are from the Progenesis image analysis results.

Figure 2 shows the 2-DE average gel representative of the RT112 parental cell line. The inserts provide a view of the expression pattern and the relative abundance of the proteins whose expression was significantly different in the parental cells and both 4-ABP-resistant clones.

Image analysis detected a comparable number of spots in the three average gels from the three cell lines (spot number 1015 ± 40, mean ± SD). The number of spots detected in each set of three replicates was reproducible (coefficient of variation, CV 6–17%), as was their spot intensity (CV 15–29%). Spots showing the highest variation were generally those not completely resolved from surrounding proteins, or localized at the extreme pI values.

Overall, fourteen protein species showed a statistically significant change in abundance of 1.5 times or more, as a result of the cell line phenotype. Results of identifications are summarized in Table 1.

**Table 1:** Proteins identified by LC-MS/MS, showing different expression levels in the different cell lines. Detailed information on peptides/proteins identification is reported in Additional File 2.

Spot #	Protein identified	Symbol	Swiss-Prot	pI Theor./Exp.^b)^	MW (kD) Theor./Exp.^b)^	#pept. ^c)^	Cov% ^d)^	Score ^e)^
46	Programmed cell death 6-interacting protein	PDCD6IP	Q8WUM4	6.13/6	96/95	15	17	93.9
146	Calreticulin	CALR	P27797	4.3/4.3	48/60	36	62	187.0
206	Aldehyde dehydrogenase 1A3 **a**	ALDH1A3a	P47895	6.9/6.4	56/56	20	25	108.6
207	Aldehyde dehydrogenase 1A3 **b**	ALDH1A3b	P47895	6.9/7	56/56	19	28	123
349	Elongation factor Tu	TUFM	P49411	7.3/7.5	49/46	12	30	64.2
431	3-Hydroxyisobutyl Coenzyme A hydrolase	HIBCH	Q53GA8	8.4/7.4	43/43	16		
463	Glyceraldehyde-3-phosphate dehydrogenase	GAPDH	P04406	8.6/8	36/37	10	27	57.1
479	Annexin A2	ANXA2	P07355	7.6/8.7	38/37	11	33	81.5
680	Transgelin-2	TAGLN2	P37802	8.4/5.8	22/21	3	19	25.1
744	Fatty acid-binding protein	FABP4	P15090	6.8/6	14/14	39	73	145.9
801	Lamin-A/C	LMNA	P02545	6.6/6.2	74/75	35	41	197.6
841	94 kDa glucose-regulated protein	GRP94	P14625	4.7/4.7	92/95	15	18	105.2
906	NADH-ubiquinone oxidoreductase 30 kDa subunit	NDUFS3	O75489	6.9/5	30/28	10	46	84.3
912	Triosephosphate isomerase	TPI	P60174	6.5/6	26/27	12	42	77.1

^a) ^Arbitrary label assigned to the different isoforms.

^b) ^Theor.: theoretical, data-based annotations; Exp.: experimental, from 2-D gels.

^c) ^# pept.: number of valid peptide matches found for the given protein

^d) ^Cov%: the percent ratio of all amino acids from valid peptide matches to the total number of amino acids in the protein.

^e) ^Score: the protein score is a function calculated from the individual normalized z-scores of validated peptides. Peptide z-score refers to the distribution of calculated scores compared to that of random peptide sequences in order to find the mean and variance . Database redundancy is handled and solved by the Phenyx software. If a protein shares all its validated peptides with another one, it is considered a subset and will not appear in the best-scoring protein list. It appears in the protein Details panel under Subset for the principal and better scoring parameters. Therefore the entries reported (Swiss_Prot AC) refer exclusively to the best-scoring protein found by the search engine.

The difference on 2-DE gel between expected and observed MW of calreticulin (CALR, spot 146) (Table 1) comes as no surprise, since the electrophoretic mobility of CALR is known to be anomalous, with the 46-kD protein showing 2-DE migration compatible with a molecular size of 60-kD [7].

Some of the proteins such as annexin 2 (ANXA2, spot 479), transgelin 2 (TANGL2, spot 680) and NADH-ubiquinone oxidoreductase 30 kDa subunit (NDUFS3, spot 906) showed a difference between the predicted and observed pI value (Table 1). The most straightforward interpretation of these shifts is the presence of protein variants differing in charge through aminoacid differences or post-translational modifications (PTM). As regards ANXA2, a slightly longer isoform of ANXA2 with a more basic pI (8.7) but no real change in the MW has been identified in our MS/MS search using another database, the NCBInr (v.20060828) (ANXA2, isoform 1, NP_001002858, # valid peptide match 11, coverage 31%). This might therefore be the variant we found in our 2-DE gel. Unfortunately, no information was found available about its biological relevance.

A multiple database (UniProt_Swiss Prot and NCBInr) search strategy did not help find any further variant of TANGL2 and NDUFS3 that might explain the pI variation observed. However, both TANGL2 and NDUFS3 presented a pI shifted toward the acidic region of the 2-DE gel, suggesting that some post-translational modifications (PTM) such as phosphorylation had occurred. These proteins have multiple phosphorylation sites, as predicted by bioinformatics tools such as NetPhos [8]. The presence of such PTM can shift the pI toward an acidic value; consequently the modified proteins might migrate differently on the 2-DE gels, as predicted by ProMoST [9], a web-tool that calculates the effect of single/multiple modifications of a protein and provides the graphic representation of the gel shifts due to PTM.

### Protein expression profile in 4-ABP-resistant clones and the RT112 parental urothelial cell line

As shown in Figure 2, histogram inserts, in the resistant cells the expression levels of ANXA2, 94 kDa glucose-regulated protein (GRP94) and lamin A/C (LMNA) were twice or more of the parental line. Five proteins were down regulated in both resistant clones. The abundance of the mitochondrial elongation factor Tu (TUFM) was more than halved compared to the parental line. Similarly, the expression level of 3-hydroxyisobutyryl-CoA hydrolase, isoform 1 (HIBCH), triosephosphate isomerase (TPI) and adipocyte fatty acid-binding protein (FABP4) was more than two times lower in the RT5 clone than in the RT112 cells. In the RT11 clone, the abundance of these proteins did not decrease two fold. Significant down regulation of NDUFS3 was also observed in the resistant cells, where decreases were 1.7 and 1.3-fold.

### Protein expression differences between the 4-ABP-resistant clones

A number of proteins were differently expressed only in the clone RT5 (Figure 2, histogram inserts). The most striking aspect was the marked decrease in the expression of TAGNL2, whose abundance in the RT5 cells was 5 times lower than in the RT11 and 6.7 times lower than the parental line. The pattern of expression was similar for glyceraldehyde 3-phosphate dehydrogenase (GAPDH), whose level was significantly lower in the RT5 clone. This clone showed a decrease in the expression of CALR, but the difference was significant only compared to the parental cells.

Three protein species were significantly down regulated only in clone RT11 compared to clone RT5 and the parental line. The abundances of the PDCD6IP and two putative isoforms of aldehyde dehydrogenase 1 family, member A3 (ALDH1A3) were about half that of the RT5 clone and the parent cell population.

### Western blot

Western blotting of lysates prepared from the parental RT112 and the 4-ABP-resistant clones RT5 and RT11 was used to investigate some of the most important factors regulating apoptosis. Among the Bcl-2 family, the balance between the Bcl-2 and the Bax members is essential to the apoptotic potential of the cells, low apoptotic activity often being associated with a low Bax/Bcl-2 ratio.

**Figure 3:**
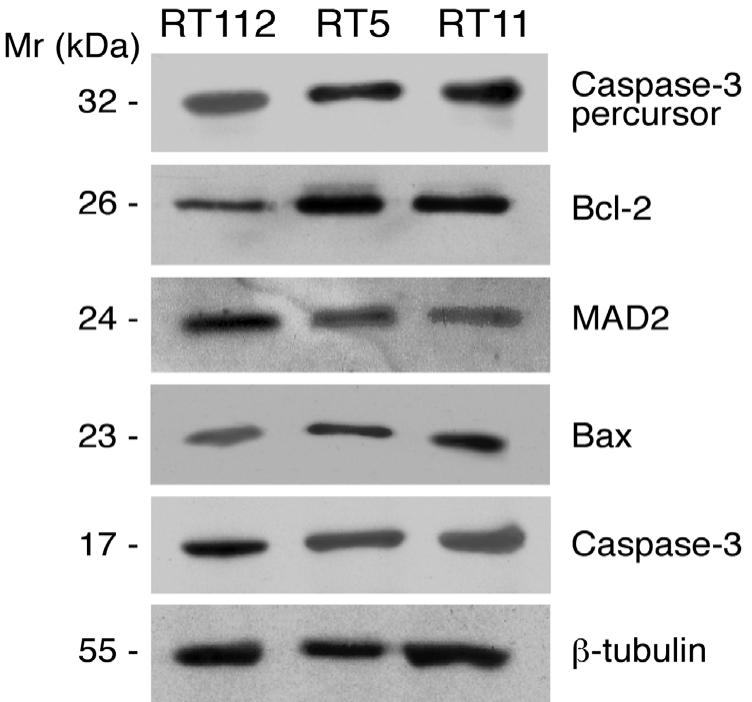
Relative changes in caspase-3, Bcl-2, Bax, Mad2 protein levels in the human bladder cancer cell line RT112 and in 4-ABP resistant clones RT5 and RT11, by Western blot analysis. Levels of β-tubulin were used as a normalizing factor for total amount of protein loaded.

As reported in figure 3, both resistant clones showed markedly higher expression of the anti-apoptotic Bcl-2 protein than the parent line. The effect on the Bax level was less evident, though the Bax/Bcl-2 expression ratio was lower in the 4-ABP resistant clone (data not shown). Consistent with the lower apoptotic ability in the resistant clones is the increased presence of inactive caspase-3 precursor (Cpp32 precursor) and the lower levels of the cleaved caspase-3 form (Cpp32), which is proteolytically generated during apoptosis (figure 3). The level of the MAD2 protein, required for mitotic checkpoint control, was also lower in the resistant clones than in the parent cells (figure 3).

## Discussion

We have successfully developed two cell lines (RT5 and RT11), from the parental human bladder cancer RT112 cell line that show resistance to the environmental carcinogen 4-ABP. Here we report the comparative proteomic study to identify the differences in abundance of proteins in the sensitive and 4-ABP-resistant RT112 urothelial cells. The aim was to gain insight into how changes in protein profiles contribute to the development of resistance to the environmental carcinogen 4-ABP. Our data as a whole provide evidence that deregulation of apoptosis might be strongly implicated in the selection of such carcinogen-resistant cells.

Although the parental RT112 cell line has been reported to have relatively low susceptibility to apoptosis [10,11], interestingly both resistant clones showed over-expression of two proteins, GRP94 and LMNA, linked to a decrease in apoptosis [12,13], suggesting that resistance to 4-ABP might be associated with a further loss of apoptotic capacity. Moreover, the apoptotic resistant phenotype of the clones might be reinforced by the down-regulation of FABP4, a fatty acid-binding protein, whose expression is linked to the apoptotic process [14,15]. In Additional file 1 we expanded upon the probable role of these proteins in conferring resistance to apoptosis.

To confirm that the observed protein changes reflected a decrease in the apoptotic response of the resistant clones, Western blot analysis on parent and resistant cell extracts was done to assess the expression levels of some key molecules related to apoptosis. The marked increased of the anti-apoptotic protein Bcl2, concomitantly with a decrease in caspase-3 activation found in the resistant clones would support the inference from proteomic analysis that the 4-ABP-resistant clones would have stronger resistance to apoptosis than their parental counterpart.

Western blot analysis also indicated that the 4-ABP-resistant clones had lower levels of MAD2 than the parental line. Down-regulation of MAD2, a component of the mitotic spindle checkpoint pathway, has been linked to inhibition of anticancer drug-induced apoptosis by up-regulating Bcl-2 [16]. Thus, our results support this functional link between the status of MAD2 and apoptosis, confirming that down-regulation of MAD2 expression would probably help cells evade mitotic arrest and apoptosis induced by the DNA-damaging agent 4-ABP.

A further finding in our resistant clones is an overall perturbation in cell metabolism and energy pathways. The expression of enzymes involved in glycolysis (TPI) oxidative phosphorylation (NDUFS3) and ketogenesis (HIBICH) was reduced, suggesting that all these pathways were somehow decelerated.

It is of interest that ANXA2, a membrane trafficking protein, was over-expressed in our resistant cells, since its up-regulation has already been reported in a doxorubicin-resistant cancer cell line [17]. So far, the physiological significance of enhanced annexin expression in resistance cells is still unclear.

Interestingly, each of the 4-ABP resistant clones showed its own expression pattern for some proteins involved in various biological processes such as cell transformation and migration (TAGNL2), glycolysis (GAPDH) and regulation of Ca^2+ ^homeostasis (CALR) [18-20]. These differences are not altogether surprising, since it cannot be excluded that cells somehow gained a selective advantage through different strategies that translate as increased carcinogen resistance. The low abundance of GAPDH and of CALR only in clone RT5 might be one of the mechanisms used by these resistant cells to halt their apoptotic response to death stimuli. Numerous studies have reported a pro-apoptotic role of GAPDH [19] and its down-regulation might point to a quenching of the apoptotic pathway, although it could also simply reflect a modulation in the rate of glycolysis. Down-regulation of CALR was recently observed in a melphalan-resistant MCF-7 breast cancer cell line [20], suggesting that it might be important in the development of resistance.

Conceivably, different 4-ABP-resistant clones might also use different molecular factors to lower their apoptotic response. This possibility might be uphold by the observation that clone RT11 had a reduced abundance of the pro-apoptotic PDCD6IP protein, but not clone RT5. This protein interacts with apoptosis-related protein such as PDCD6 and endophilin and its down-regulation may be a link to cell death resistance [21].

## Conclusion

In this study we highlighted the importance of the effects on the expression of proteins related to membrane trafficking and apoptotic pathways in conferring resistance to the carcinogen 4-ABP. These two processes have also been evoked for the development of chemoresistance [4,22,23], suggesting that they might serve as signatures for cardinal mechanisms by which cells develop resistance to the action of cytotoxic drugs and probably to carcinogens, so they could be potential biomarkers of resistance and cancer risk.

It is of course not straightforward to correlate alterations in the expression of every single protein and resistance to 4-ABP. A note of caution is therefore due here. The unique expression patterns in the resistant clones may be just a phenotype of these cell populations and may make no direct contribution to resistance. It is still not clear whether the resistant clones are the result of a transformation of the RT112 cell line by 4-ABP or of a selection of subclones inherently present among RT112 cells.

## Materials and methods

### Cell cultures

The human papillary non-metastatic bladder cancer cell line RT112 was kindly provided by Prof. Alberto Bardelli (Institute for Cancer Research and Treatment, IRCC, Candiolo, Torino, Italy). Cells were cultured in RPMI 1640 medium supplemented with 10% (v/v) FBS, 1% penicillin-streptomycin solution (10.000 units penicillin-G and 10 mg streptomycin per mL) and 1% L-glutamine solutions (2 mM). Media components were all from Sigma (Italy).

### Carcinogen treatment and development of resistant cell lines

4-ABP was dissolved in DMSO (Sigma) and activated in NADPH generating system solution (0.27 mM NADP, 5 mM glucose 6-phosphate, 0.45 mM MgCl_2_, 0.45 mM KCl and 200 mM Tris-HCl, Sigma) containing 0.4 mg/mL Aroclor 1254-induced S9 rat liver extract (BD Italy) for 2 h at 37°C. Cells were treated with the carcinogen for 4 h at 37°C then washed and resuspended in complete medium. Resistant cells were established by two treatments with 125 ng/mL 4-ABP, the concentration causing 99% death of RT112 cells. Treatment was repeated after cells surviving the first round had recovered exponential growth. Single-cell clones were obtained by limiting dilution. The relative resistance of isolated clones was evaluated by treating 5 × 10^5 ^cells with appropriate concentrations of the carcinogen ranging from 75 to 175 ng 4-ABP/mL. Relative resistance of the treated cultures was normalized to the plating efficiency of the untreated controls. The full development and characterization of the resistant cell lines will be reported in a forthcoming publication (F. Saletta et al., submitted).

### Cell lysis and protein solubilization for 2-DE

Cells at 80% confluent were harvested by scraping, washed three times with ice-cold 10 mM PBS and pelleted. Pellets (5 × 10^6 ^cells/pellet) were shock-frozen in liquid nitrogen and stored at -80°C until further processing. Cell lysis and protein extraction was done in a solution containing 5 M urea, 2 M thiourea, 2% CHAPS, 2% Zwittergent and a mixture of protease inhibitors (complete, mini EDTA-free cocktail, Roche). DeStreak reagent (100 mM) (GE Healthcare, Italy) was added in order to protect cysteinyl groups and prevent non-specific oxidation. The suspension was incubated for 40 minutes at 4°C on a mixing wheel. Cell debris was removed by centrifugation at 15,000 × g for 15 min, then the supernatant was aliquoted and stored at -80°C. Protein concentration was determined using the PlusOne 2-D Quant kit (GE Healthcare, Italy).

### 2-DE

For each gel, 300 μg of total protein were dissolved to a final volume of 250 μL in the re-hydration solution (5 M urea, 2 M thiourea, 2% CHAPS, 2% Zwittergent, 100 mM DeStreak and 0.5% IPG buffer pH 3–10 linear, GE Healthcare, Italy), and then applied on immobilized pH 3–10 linear gradient strips (IPG strip, GE Healthcare, Italy). The strips were hydrated on an IPGphor apparatus (GE Healthcare, Italy) for 16 h at 30 V/h, then focused for 26 h until 50,000 Vhr. After the first-dimension run, proteins were reduced by incubating individual strips for 15 min in a solution containing 50 mM Tris-HCl pH 8.8, 6 M urea, 30% glycerol, 2% SDS, 60 mM dithiothreitol (DTT, GE Healthcare, Italy). Proteins were then alkylated by incubating the strips for 15 min in a similar solution, with DTT replaced by 100 mM iodoacetamide. The strips were embedded in 0.7% (w/v) agarose on the top of 1 mm-thick acrylamide gels cast at 7.5–17.5%. Proteins were separated by mass by electrophoresis at 10 mA/gel. This was done overnight, at 4°C, in a running buffer composed of 25 mM Tris, 250 mM glycine, 0.1% SDS. Gels were rinsed three times with de-ionised H_2_O, fixed for 1 hour in an aqueous solution with 50% methanol and 7% acetic acid, and rinsed again with de-ionised H_2_O. Finally, gels were stained with colloidal Coomassie Blue (Pierce) for 4–5 hours then extensively washed with de-ionised H_2_O [24].

Three replicate gels were run for each experimental condition (parental cell lines and two resistant clones).

Stained gels were scanned at 16-bit resolution (Expression 1680 Pro, Epson) and the resulting TIFF images were analysed with Progenesis Workstation software (v2005, Nonlinear Dynamics, UK). The Progenesis automatic analysis protocol for the images of the nine gels included spot detection, warping, background subtraction, average gel creation, matching and reference gel modification. Spot volumes were normalized against the total volume of all the spots in the gel. Average gels were generated by the software for spot pattern comparison. They are a statistical combination of the gels in a group, showing mean spot values with the associated error, providing information about spot variability within the gel set. An average gel was created for each experimental group by combining the three replicates. The criterion for including a spot in the average gel was that any spot must be present in all replicates. Spot editing (spot splitting corrections and match editing) was done sparingly, and only on selected complex areas of the gel.

### Statistical analysis

Statistical comparisons of the individual protein abundance in the three cell clones (one-way ANOVA) and between-groups comparisons (multiple comparison test, Tukey Kramer HSD, p < 0.05) were computed using JMP v6 software (SAS Institute Inc.).

### Protein identification by mass spectrometry

In-gel digestion was done as previously described [24]. Briefly, the spots of interest were excised manually from the gel and digested with sequencing-grade modified trypsin. Aliquots of the supernatant containing tryptic peptides were directly analysed by mass spectrometry.

Liquid chromatography (reverse-phase microbore-LC)-tandem mass spectrometry (LC-MS/MS) was done as previously reported [24] using a Surveyor system (autosampler and MS pump) coupled to an ion-trap mass spectrometer LCQ Deca XP^Plus ^(Thermo Finnigan) equipped with a standard electrospray source, operated in positive ion mode, with an ion sprayer voltage of 4.6 kV and capillary temperature of 220°C.

Data were acquired sequentially in MS mode (scan range of 450–2000 amu), and in data-dependent mode, recording the MS/MS spectra of the three most intense ions of each MS scan. The MS/MS spectra were acquired with an isolation width of 3.0 amu and normalized collision energy of 45%. Raw MS/MS data from each LC run were transformed into dta files using the instrument software (BioWorks, rev. 3.1 SR1), with automatic selection of individual MS/MS spectra.

Tandem mass spectra were analysed using the MS/MS search engine Phenyx version 1.9 (GenBio, Switzerland) against the UniProt_Swiss Prot database (version 50.7).

The search was enzymatically constrained for trypsin, and allowed for one missed cleavage site. Further search parameters were: no restriction on molecular weight (MW) and isoelectric point; taxonomy: *Homo sapiens*; fixed modification: carbamidomethylation of cysteine; variable modification: oxidation of methionine.

A summary table is available (see Additional file 2) that concisely restates the main submission parameters including algorithm, scoring models, thresholds. All information concerning peptide identification is available in Additional file 3, derived from the Phenyx Database/AC/Peptide view results page.

### Western blotting

The electrophoretic pattern of proteins related to apoptosis (Bax, Bcl-2, caspase-3) and mitotic checkpoints (mitotic arrest deficient 2, MAD2) was investigated by Western blot analysis. Cells extracts were prepared by lysing cells in the lysis buffer (Triton X-100, 1 M Tris, 5 M NaCl) in the presence of aprotinin, leupeptine and PMSF as protease inhibitors, for 30 minutes on ice. Insoluble materials was pelletted at 13,000 × g for 10 minutes at 4°C and the protein concentration was determined using a Biorad assay kit (BioRad, Milan, Italy).

Total cellular proteins (30 μg protein/lane) were separated on SDS-10% polyacrylamide resolving gels using the Mini Protean II electrophoresis system at 100 V, for 2 hours (Bio-Rad, Milan, Italy). Proteins were transferred to nitrocellulose transfer membrane (Whatman, UK) using the transfer buffer (50 mM Tris, 100 mM glycine, SDS 0.01%, 20% methanol) and the Bio-Rad Trans-blot system (55 V, 2 h).

Blots were rinsed with TBS-T buffer (10 mM Tris-HCl pH 8, 150 mM HCl, 0.05% v/v Tween-20) and blocked in TBS-T, 5% w/v non-fat dried milk (Nestlé, Italy) for 2 h.

After overnight incubation with primary antibody diluted 1:300 in TBS-T, 5% non-fat dried milk, (rabbit polyclonal antibodies: β-tubulin H-235, caspase-3 H-277, MAD2 FL-205, Bcl-2 N-19, Bax N-20; mouse monoclonal antibody caspase-3 E-8; Santa Cruz Biotechnology, USA) blots were washed with TBS-T and incubated with secondary antibody at 1:1000 for 2 h. Peroxidase-conjugated anti-mouse or anti-rabbit IgG HRP (Santa Cruz, Biotechnology, USA) were used as secondary antibodies.

Blots were revealed using enhanced chemiluminescence (ECL) (GE, Milan, Italy) and scanned as 16-bit images (Expression 1680 Pro, Epson). The resulting TIFF images were analysed using the Progenesis software (v2005, Nonlinear Dynamics, U.K, Nonlinear Dynamics, UK). Expression data were normalized relative to β-tubulin.

## Abbreviations

2-DE: bi-dimensional electrophoresis

4-ABP: 4-aminobiphenyl

ALDH1A3, aldehyde dehydrogenase 1 family, member A3;

ANXA2, annexin 2;

CALR, calreticulin;

Cpp32 precursor, caspase-3 precursor;

Cpp32, caspase-3 active form;

FABP4, fatty acid-binding protein, adipocyte;

GAPDH, glyceraldehyde 3-phosphate dehydrogenase;

GRP94, 94 kDa glucose-regulated protein;

HIBCH, 3-hydroxyisobutyryl-CoA hydrolase, isoform 1;

LMNA, lamin A/C;

MAD2, mitotic arrest deficient 2;

NDUFS3, NADH-ubiquinone oxidoreductase 30 kDa subunit, mitochondrial;

PCD6IP, programmed cell death 6 interacting protein;

TAGLN2, transgelin-2;

TPI, triosephosphate isomerase;

TUFM, elongation factor Tu, mitochondrial.

## Competing interests

The author(s) declare that they have no competing interests.

## Authors' contributions

RP planned and supervised proteomic analysis, did data mining, interpretation of data and prepared the manuscript.

LA conceived the study and critically reviewed the results and the manuscript.

DC, RC, CdO and SS performed proteins extraction, two-dimensional electrophoresis, western blots, mass spectrometry and data handling.

FS developed the resistant cell lines.

PV and GM initiated the carcinogen cell lines resistant study developing the concept, and contributed to the preparation of the manuscript.

All authors read and approved the final manuscript.

## Supplementary Material

Additional file 1 Expanded discussion. Expanded discussion upon the probable role of some proteins in conferring resistance to apoptosis as outlined in the main article. Click here for file

Additional file 2 Phenyx submission parameters. Summary of the main MS/MS submission parameters including algorithm, scoring models, thresholds. Click here for file

Additional file 3 Protein identification by Phenyx. Information concerning peptide identification as derived from Phenyx Database/AC/Peptide view results page Click here for file
