# Supplementary material for: Proteome characterization of a human urothelial cell line resistant to the bladder carcinogen 4-aminobiphenyl
Source: Proteome Sci. 2007 May 3;5:6. doi: 10.1186/1477-5956-5-6 (PMC1871571; doi:10.1186/1477-5956-5-6)
Supplement: Additional file 1 — Expanded discussion. Expanded discussion upon the probable role of some proteins in conferring resistance to apoptosis as outlined in the main article. [file 1477-5956-5-6-S1.pdf]

Additional file 1

Expanded discussion

In this material, we discuss the possible role that three proteins might have in conferring the apoptotic resistance phenotype to both 4-ABP resistant clones RT5 and RT11 as outlined in the main article.

94kDa glucose-regulated protein; GRP94

GRP94 is an oxidative stress-induced endoplasmic reticulum chaperone with Ca^{2+} binding properties and a component of the unfolded protein response (UPR). It may have an antiapoptotic effect and suppress oxidative apoptosis, probably protecting cells against flux in Ca^{2+} homeostasis, which could result in cell death [1]. Thus, the constitutive increase of GRP94 at the protein level in our resistant cell populations might contribute to their resistance to apoptosis.

Lamin A/C; LMNA

Lamin A/C is an intermediate filament type-V protein, component of the nuclear lamina, a filamentous protein network underlying the inner nuclear membrane.

Reduction of this protein by proteolytic degradation (caspase-dependent) has been recognized as a prelude to nuclear destruction in apoptosis [2].

It is possible to hypothesise that LMNA increased expression in RT5 and RT11 *versus* the parental RT112 might be an additional marker of the possibly lower apoptotic potential in the resistant clones.

Fatty acid-binding protein, adipocyte; FABP4

FABP4 is one of the fatty acid-binding proteins and plays a role in intracellular lipid transport and metabolism as well as in signal transduction [3]. FABPs proteins are

probably involved in the fatty acid-mediated apoptotic process *via* shuttling their ligands within cells. It has been recently shown that over-expression of FABP4 causes apoptosis in prostate cancer cells, and regulates cell proliferation via enhanced TNFalpha expression, suggesting the involvement of FABP4 in the gene expression regulation in the apoptotic process. [4]. Moreover apoptosis-linked enhancement of FABP4 has been reported in murine lymphocytes in response to dexamethasone treatment [5].

Therefore, the lower abundance of FABP4 might render our cells more resistant to apoptosis in response to 4-ABP exposure.

References

1. Bando Y, Katayama T, Kasai K, Taniguchi M, Tamatani M, Tohyama M: **GRP94 (94 kDa glucose-regulated protein) suppresses ischemic neuronal cell death against ischemia/reperfusion injury**. *Eur J Neurosci* 2003, **18**: 829-840.
2. Burke B: **Lamins and apoptosis: a two-way street?** *J Cell Biol* 2001, **153**: F5-7.
3. Glatz JF, Borchers T, Spener F, van der Vusse GJ: **Fatty acids in cell signalling: modulation by lipid binding proteins**. *Prostaglandins Leukot Essent Fatty Acids* 1995, **52**: 121-127.
4. De Santis ML, Hammamieh R, Das R, Jett M: **Adipocyte-fatty acid binding protein induces apoptosis in DU145 prostate cancer cells**. *J Exp Ther Oncol* 2004, **4**: 91-100.
5. Abdelwahab SA, Owada Y, Kitanaka N, Adida A, Sakagami H, Ono M, Watanabe M, Spener F, Kondo H: **Enhanced expression of adipocyte-type fatty acid binding protein in murine lymphocytes in response to dexamethasone treatment**. *Mol Cell Biochem* 2006. DOI:10.1007/s11010-005-9050-1
