# Supplementary material for: Proteome characterization of a human urothelial cell line resistant to the bladder carcinogen 4-aminobiphenyl
Source: Proteome Sci. 2007 May 3;5:6. doi: 10.1186/1477-5956-5-6 (PMC1871571; doi:10.1186/1477-5956-5-6)
Supplement: Additional file 2 — Phenyx submission parameters. Summary of the main MS/MS submission parameters including algorithm, scoring models, thresholds. [file 1477-5956-5-6-S2.pdf]

Additional file 2. Phenyx submission parameters

**Scoring model**	ESI - ion trap (LCQ)	
**Parent charge**	1,2,3 (trust=yes)	
**Rounds**	**1**	**2**
**Modifications**	Cys_CAM[fixed, all]	Cys_CAM[fixed, all]
	Oxidation_M[variable, none]	Oxidation_M[variable, none]
**Enzyme**	Trypsin_(KR_noP)	Trypsin_(KR_noP)
	miss. cleav.=1	miss. cleav.=2
	cleavMode=normal	cleavMode=half cleaved
**AC score**	6	6
**Pept thresholds**	score>=5.0	score>=4.0
	p-value<=1.0E-4	p-value<=0.0010
	length>=6	length>=6
**Conflict resolution**	none	yes
**Parent tol.**	1.0Da	2.0Da
**Turbo scoring**	tolerance=800.0ppm	no
	coverage >=0.2	
	series=b;b++;y;y++	

Scoring model: refers to the algorithm that generates the score between experimental and theoretical masses, depending on the type of instrument.

Parent charge: refers to the charge state of the parent ions, singly-, doubly-, triply-charged

Rounds: sets of calculations on the data. A second round of calculations is used to fine-tune the results from the first round of scoring because only the accession numbers that fulfilled the first round criteria are processed during the second round.

Modifications: fixed chemical/post-translational modification that occurs for every instance of the modifiable amino acid in the protein sequence; variable modification that may or may not occur.

Enzyme: the enzyme used to digest the protein. The error allowance for enzyme inefficiency is included as number of sites per peptide that were not cut (Miss Cleav) and as occurrence of the digestion according to the cleavage rules (one or both ends of protein) (CleavMode).

AC score: refers to the minimum significant value for a protein's accession number score. Protein matches scoring lower than this value (set at 6) are rejected from the identified proteins.

Peptide thresholds: refer to three parameters *(i) minimum peptide z-score*, the minimum distance from a random match; *(ii) minimum peptide p-value*, the probability of a peptide match in a database occurring by chance with the associated z-score or better. The lower the p-value, the more significant the match; *(iii) minimum peptide length*, peptides with less than the specified number of amino acids (6) are reported in the peptide match results but do not contribute to the protein score.

Conflict resolution: conflicts arise when the scoring algorithm can match more than one peptide with an acceptable z-score and p-value to a given spectrum. Phenyx resolves a conflict only if the

conflicting peptide matches are of good enough quality, i.e. if they reach a minimum z-score and p-value. These thresholds are a function of the parent charge and are set by Phenyx. If the z-scores and p-values are too low, then the conflict is not resolved and the matches are rejected.

Parent tolerance: parent error tolerance, the deviation allowed between experimental (observed) parent ion masses and the theoretical (calculated) masses.

Turbo scoring: a procedure that accelerates the searches by pre-processing the data before submitting them to the main scoring calculation. A minimum percentage (20%) of the peptide sequence coverage by b^+(b), b^{2+} (b^{++}), y^+ (y) or y^{2+} (y^{++}) fragment series is looked for. If this percentage is not attained, the spectrum is not submitted for further scoring.

In-depth explanations of each descriptor are available at www.phenyx-ms.com/.
