# Supplementary material for: Proteome characterization of a human urothelial cell line resistant to the bladder carcinogen 4-aminobiphenyl
Source: Proteome Sci. 2007 May 3;5:6. doi: 10.1186/1477-5956-5-6 (PMC1871571; doi:10.1186/1477-5956-5-6)
Supplement: Additional file 3 — Protein identification by Phenyx. Information concerning peptide identification as derived from Phenyx Database/AC/Peptide view results page [file 1477-5956-5-6-S3.pdf]

Additional file 3

Protein identification by Phenyx

spot #	Identified protein	Symbol	Swiss-Prot #	#pept.	Cov%	Score	sequence	charge	m/z	d m/z	z-score	P-value	Pos.	#MC	Modif
spot 46	Programmed cell death 6-interacting protein	PDCD6IP	Q8WUM4	15	17	93.9	K/FLTALAQDGVINEEALSVTELDR/V	2	1253.304	-0.656	11.4	4.32E-27	584-606	0	::::::::::::::::::::::::
							K/LANQAADYFGDAFK/Q	2	767.464	-1.599	10.7	4.38E-23	216-229	0	:::::::::::::::
							K/LANQAADYFGDAFK/Q	2	765.844	0.021	10.1	3.07E-20	216-229	0	:::::::::::::::
							R/SVIEQGGIQTVDQLIK/E	2	864.594	-0.113	9.88	1.59E-19	423-438	0	:::::::::::::::::
							K/FIQQTYPSGGEEQAQYCR/A	2	1081.974	-0.49	9.7	7.51E-19	24-41	0	:::::::::::::::::Cys_CAM::
							K/LALASLGYEK/S	2	532.929	-0.126	9.57	5.11E-18	111-120	0	:::::::::::
							K/SCVLFNCAALASQIAAEQNLDNDEGLK/I	3	985.109	-0.642	9.21	9.09E-17	121-147	0	::Cys_CAM:::::Cys_CAM:::::::::::::::::::::
							K/SLLSNLDEVK/K	2	559.359	-0.05	8.32	4.24E-13	554-563	0	:::::::::::
							K/LANQAADYFGDAFK/Q	2	764.319	1.546	7.84	1.65E-11	216-229	0	:::::::::::::::
							R/YYDQICSIEPK/F	2	708.384	-0.055	7.32	9.68E-10	71-81	0	::::::Cys_CAM::::::
							K/FIQQTYPSGGEEQAQYCR/A	3	721.969	-0.644	6.9	2.12E-08	24-41	0	:::::::::::::::::Cys_CAM::
							K/KTSEVDLAKPLVK/F	3	476.659	-0.039	6.26	1.93E-06	11-23	1	::::::::::::::
							R/SVIEQGGIQTVDQLIK/E	3	576.789	-0.133	6.13	4.00E-06	423-438	0	:::::::::::::::::
							K/ELPELLQR/N	2	499.179	0.109	5.65	7.87E-05	439-446	0	:::::::::
							K/SLLSNLDEVKK/E	2	623.609	-0.253	5.59	0.000107	554-564	1	::::::::::::
spot146	Calreticulin	CALR	P27797	36	62	187	F/LITNDEAYAEEFGNETWGVTK/A	3	796.839	-0.466	11.5	5.37E-27	314-334	half	::::::::::::::::::::::
							T/NDEAYAEEFGNETWGVTK/A	2	1030.634	-0.186	10.6	1.10E-22	317-334	half	:::::::::::::::::::
							T/NDEAYAEEFGNETWGVTK/A	3	687.524	-0.223	9.96	9.42E-20	317-334	half	:::::::::::::::::::
							K/SGTIFDNFLIT/N	2	614.369	-0.052	9.89	2.19E-19	306-316	half	::::::::::::
							E/EFGNETWGVTK/A	2	634.379	-0.077	9.22	1.24E-16	324-334	half	::::::::::::
							K/HEQNIDCGGGYVK/L	2	739.374	-0.543	9.22	1.16E-16	82-94	0	:::::::Cys_CAM:::::::
							K/FYGDEEKDKGLQTSQDAR/F	3	696.534	-0.207	8.84	3.87E-15	39-56	2	:::::::::::::::::::
							K/EQFLDGDGWTSR/W	2	706.194	-0.376	8.8	5.27E-15	8-19	0	:::::::::::::
							K/IDNSQVESGSLEDDWDFLPPK/K	2	1196.864	-0.811	8.48	2.49E-14	169-189	0	::::::::::::::::::::::
							K/IKDPDASKPEDWDER/A	2	901.099	-0.175	7.63	7.37E-11	191-205	1	::::::::::::::::
							K/HEQNIDCGGGYVK/L	3	492.644	0.245	7.5	3.21E-10	82-94	0	:::::::Cys_CAM:::::::
							T/NDEAYAEEFGNETWGVTK/A	2	1030.959	-0.511	7.49	1.87E-10	317-334	half	:::::::::::::::::::
							R/CKDDEFTHLY/T	2	664.489	-0.204	7.19	2.70E-09	146-155	half	:Cys_CAM::::::::::
							L/SASFEPFSNK/G	2	557.444	-0.18	7.11	5.54E-09	61-70	half	:::::::::::
							K/IDNSQVESGSLEDDWDFLPPKK/I	3	840.714	-0.311	7.05	6.00E-09	169-190	1	:::::::::::::::::::::::
							K/IDNSQVESGSLEDDWDFLPPK/K	2	1196.594	-0.541	7.05	2.03E-09	169-189	0	::::::::::::::::::::::
							K/IDNSQVESGSLEDDWDFLPPK/K	3	798.054	-0.35	7.02	7.27E-09	169-189	0	::::::::::::::::::::::
							N/SLDQTDMHGDSEYNIMFGPDICGPGTK/K	3	1006.739	-0.314	7	7.13E-09	99-125	half	:::::::Oxidation_M:::::::::Oxidation_M::::::Cys_CAM::::::
							F/GNETWGVTK/A	2	496.319	-0.073	6.81	5.03E-08	326-334	half	::::::::::
							M/FGPDICGPGTK/K	2	574.909	-0.135	6.77	5.97E-08	115-125	half	::::::Cys_CAM::::::
							K/IDNSQVESGSLEDDWDFLPPK/K	2	1196.349	-0.296	6.7	2.45E-08	169-189	0	::::::::::::::::::::::
							R/QIDNPDYK/G	2	496.979	-0.241	6.68	1.23E-07	262-269	0	:::::::::
							K/GLQTSQDAR/F	2	488.529	-0.282	6.59	2.43E-07	48-56	0	::::::::::
							K/IKDPDASKPEDWDER/A	3	601.229	-0.278	6.55	2.76E-07	191-205	1	::::::::::::::::
							Y/TLIVRPDNTYEVK/I	2	774.979	-0.554	6.38	6.55E-07	156-168	half	::::::::::::::
							K/IDNSQVESGSLEDDWDFLPPK/K	2	1196.434	-0.381	6.27	4.05E-07	169-189	0	::::::::::::::::::::::
							K/IDNSQVESGSLEDDWDFLPPK/K	3	798.054	-0.35	6.24	1.50E-06	169-189	0	::::::::::::::::::::::
							Y/TLIVRPDNTYEVK/I	3	517.019	-0.4	6.04	8.20E-06	156-168	half	::::::::::::::
							K/LFPNSLDQTDMHGDSEYNIM/F	2	1172.904	-0.905	6.04	1.82E-06	95-114	half	:::::::::::Oxidation_M::::::::::
							L/IVRPDNTYEVK/I	2	667.849	-0.49	5.99	9.25E-06	158-168	half	::::::::::::
							K/HEQNIDCGGGYVK/L	3	493.044	-0.155	5.69	6.53E-05	82-94	0	:::::::Cys_CAM:::::::
							T/DSKPEDWDKPEHIPDPDAK/K	3	740.719	-0.372	5.68	5.02E-05	213-231	half	::::::::::::::::::::
							-1/EPAVYFK/E	1	853.59	-0.145	5.52	3.39E-05	1-07	0	::::::::
							Y/FKEQFLDGDGWTSR/W	3	562.824	-0.222	5.4	0.000304	6-19	half	:::::::::::::::
							K/GTWIHPEIDNPEYSPDPS/I	2	1027.984	-0.523	5.39	0.000191	270-287	half	:::::::::::::::::::
							K/KPEDWDEEMDGEWEPPVIQNPEYK/G	3	993.384	-0.616	5.24	0.000457	232-255	0	:::::::::Oxidation_M::::::::::::::::
spot 206	Aldehyde dehydrogenase 1A3 **a**	ALDH1A3a	P47895	20	25	108.6	R/ANSTDYGLTAAVFTK/N	2	780.134	-0.243	10.5	2.76E-22	432-446	0	::::::::::::::::
							R/VFVEEQVYSEFVR/R	3	544.594	-0.319	9.87	2.76E-19	320-332	0	::::::::::::::
							K/TIPTDDNVVCFTR/H	2	769.524	-0.154	8.96	1.22E-15	155-167	0	::::::::::Cys_CAM::::
							K/FATCNPSTR/E	2	527.299	-0.056	8.26	6.61E-13	49-57	0	::::Cys_CAM::::::
							R/VFVEEQVYSEFVR/R	2	816.254	-0.345	8.08	2.20E-12	320-332	0	::::::::::::::
							R/ELGEYALAEYTEVK/T	3	539.039	-0.104	7.83	2.56E-11	488-501	0	:::::::::::::::
							K/IAFTGSTEVGK/L	2	555.174	0.122	7.75	4.20E-11	253-263	0	::::::::::::
							R/YFAGWADK/I	2	479.329	-0.102	7.58	1.88E-10	143-150	0	:::::::::
							I/PTDDNVVCFTR/H	2	662.764	-0.46	7.37	7.29E-10	157-167	half	::::::::Cys_CAM::::
							K/AVEAAQVAFQR/G	2	596.169	-0.849	7.27	1.72E-09	74-84	0	::::::::::::
							K/IAFTGSTEVGK/L	2	555.534	-0.238	7.17	3.35E-09	253-263	0	::::::::::::
							K/IFINNEWHESK/S	3	473.174	-0.272	7.15	4.78E-09	34-44	0	::::::::::::
							K/EEIFGPVQPILK/F	2	685.654	-0.264	7.08	5.63E-09	410-421	0	:::::::::::::
							K/IFINNEWHESK/S	2	709.099	-0.25	7.06	6.63E-09	34-44	0	::::::::::::
							K/ILELIESGKK/E	2	565.369	-0.024	6.71	9.36E-08	365-374	1	:::::::::::
							K/ILELIESGK/K	2	501.649	-0.351	6.22	2.73E-06	365-373	0	::::::::::
							R/ELGEYALAEYTEVK/T	2	808.209	-0.31	6.21	1.81E-06	488-501	0	:::::::::::::::
							K/AVEAAQVAFQR/G	2	596.064	-0.744	5.57	0.000123	74-84	0	::::::::::::
							R/ANSTDYGLTAAVFTK/N	3	520.564	-0.301	5.52	0.000164	432-446	0	::::::::::::::::
							R/IAKEEIFGPVQPILK/F	3	561.854	-0.52	5.45	0.00024	407-421	1	::::::::::::::::
spot 207	Aldehyde dehydrogenase 1A3 **b**	ALDH1A3b	P47895	19	28	123	I/PTDDNVVCFTR/H	2	662.454	-0.15	10.6	1.34E-22	157-167	half	::::::::Cys_CAM::::
							R/ANSTDYGLTAAVFTK/N	2	780.154	-0.263	10.5	4.26E-22	432-446	0	::::::::::::::::
							K/FATCNPSTR/E	2	527.354	-0.111	9.68	1.79E-18	49-57	0	::::Cys_CAM::::::

Additional file 3

Protein identification by Phenyx

							K/TIPTDDNVVCFTR/H	2	769.614	-0.244	9.51	6.74E-18	155-167	0	::::::::::Cys_CAM::::
							R/VFVEEQVYSEFVR/R	2	816.574	-0.665	9.42	1.60E-17	320-332	0	::::::::::::::
							R/VFVEEQVYSEFVR/R	3	544.459	-0.184	8.98	1.33E-15	320-332	0	::::::::::::::
							K/IAFTGSTEVGK/L	2	555.409	-0.113	8.98	1.25E-15	253-263	0	::::::::::::
							R/ELGEYALAEYTEVK/T	2	807.964	-0.065	8.59	3.01E-14	488-501	0	:::::::::::::::
							R/ANSTDYGLTAAVFTK/N	3	520.814	-0.551	8.13	2.17E-12	432-446	0	::::::::::::::::
							R/ELGEYALAEYTEVK/T	3	539.099	-0.164	7.45	4.85E-10	488-501	0	:::::::::::::::
							K/EEIFGPVQPILK/F	2	685.799	-0.409	7.41	5.16E-10	410-421	0	:::::::::::::
							K/AVEAAQVAF/Q	2	453.354	-0.114	7.22	3.12E-09	74-82	half	::::::::::
							R/IAKEEIFGPVQPILK/F	3	561.559	-0.225	6.47	4.79E-07	407-421	1	::::::::::::::::
							R/YFAGWADK/I	2	479.334	-0.107	6.04	8.36E-06	143-150	0	:::::::::
							K/RANSTDYGLTAAVFTK/N	3	572.529	-0.232	5.84	2.60E-05	431-446	1	:::::::::::::::::
							K/SIEEVIKR/A	2	487.289	-0.001	5.68	7.82E-05	424-431	1	:::::::::
							K/IFINNEWHESK/S	3	473.064	-0.162	5.63	9.71E-05	34-44	0	::::::::::::
							K/ILELIESGKK/E	2	565.699	-0.354	5.53	0.000154	365-374	1	:::::::::::
							K/MSGNGRELGEYALAEYTEVK/T	3	745.674	-0.654	5.36	0.000299	482-501	1	:Oxidation_M::::::::::::::::::::
spot 349	Elongation factor Tu	TUFM	P49411	12	30	64.2	R/TIGTGLVTNTLAMTEEEK/N	2	954.684	-0.199	9	6.63E-16	387-404	0	:::::::::::::::::::
							K/ADAVQDSEMVELVEIR/E	2	1031.609	-0.105	8.91	1.37E-15	140-157	0	:::::::::Oxidation_M::::::::
							K/KGDECELLGHSK/N	3	458.554	-0.332	8.6	4.63E-14	243-254	1	:::::Cys_CAM::::::::
							R/AEAGDNLGALVR/G	2	593.304	0.011	7.21	2.50E-09	273-284	0	:::::::::::::
							K/ADAVQDSEMVELVEIR/E	2	1023.714	-0.207	7.01	6.26E-09	140-157	0	:::::::::::::::::
							R/ELLTEFGYK/G	2	549.984	0.303	6.95	1.83E-08	158-166	0	::::::::::
							R/TIGTGLVTNTLAMTEEEK/N	2	963.089	-0.606	6.9	1.42E-08	387-404	0	:::::::::::::Oxidation_M::::::
							R/GITINAAHVEYSTAAR/H	2	837.344	0.09	6.48	3.09E-07	62-77	0	:::::::::::::::::
							R/GTVVTGTLER/G	2	516.819	-0.031	6.28	1.81E-06	229-238	0	:::::::::::
							R/GITINAAHVEYSTAAR/H	3	558.724	-0.099	6.09	5.62E-06	62-77	0	:::::::::::::::::
							K/TTLTAAITK/I	2	460.219	0.058	5.38	0.000445	28-36	0	::::::::::
							R/IILPPEKELAMPGEDLK/F	3	637.539	-0.522	5.26	0.000655	346-362	1	:::::::::::Oxidation_M:::::::
spot 431	3-hydroxyisobutyl Coenzyme A hydrolase	HIBCH	Q53GA8	16			K/TLQEVLTMEYR/L	2	692.274	-0.421	9.53	1.18E-17	320-330	0	::::::::::::
							R/AGIATHFVDSEK/L	2	638.064	-0.241	9.38	5.42E-17	209-220	0	:::::::::::::
							S/KHTDAAEEVLLEK/K	2	742.109	-0.215	9.28	1.15E-16	29-41	half	::::::::::::::
							K/ENIASVLENYHTESK/I	2	867.384	0.037	8.99	1.37E-15	237-251	0	::::::::::::::::
							K/TLQEVLTMEYR/L	2	699.994	-0.143	8.91	3.63E-15	320-330	0	::::::::Oxidation_M::::
							K/EVTEEDLNNHFK/S	2	737.809	0.035	7.91	1.79E-11	365-376	0	:::::::::::::
							K/SFILEEHMDK/I	2	624.794	0.006	7.82	4.32E-11	257-266	0	:::::::::::
							K/SLGSSDLKF/-1	2	477.314	-0.064	7.63	2.21E-10	377-385	1	::::::::::
							K/KWEQDPETFLIIIK/G	3	587.659	-0.333	7.47	7.43E-10	73-86	1	:::::::::::::::
							K/AFCAGGDIR/V	2	483.739	-0.012	7.3	2.86E-09	92-100	0	:::Cys_CAM:::::::
							S/KHTDAAEEVLLEK/K	3	495.299	-0.368	7.15	8.81E-09	29-41	half	::::::::::::::
							R/AVLIDKDQSPK/W	2	607.574	-0.231	7.01	2.06E-08	347-357	1	::::::::::::
							K/LAMLEEDLLALK/S	2	680.074	-0.19	6.24	3.50E-06	221-232	0	:::::::::::::
							K/LAMLEEDLLALK/S	2	688.059	-0.178	6.14	6.66E-06	221-232	0	:::Oxidation_M::::::::::
							K/ENIASVLENYHTESK/I	3	578.709	-0.093	5.81	5.25E-05	237-251	0	::::::::::::::::
							K/EVTEEDLNNHFK/S	3	492.454	-0.222	5.42	0.000582	365-376	0	:::::::::::::
spot 463	Glyceraldehyde-3-phosphate dehydrogenase	GAPDH	P04406	10	27	57.1	R/VPTANVSVVDLTCR/L	2	766.094	-0.193	11.3	5.53E-26	234-247	0	:::::::::::::Cys_CAM::
							K/LISWYDNEFGYSNR/V	2	882.594	-0.189	10.6	7.57E-23	309-322	0	:::::::::::::::
							K/IISNASCTTNCLAPLAK/V	2	918.149	-0.685	10.3	3.11E-21	145-161	0	:::::::Cys_CAM::::Cys_CAM:::::::
							K/WGDAGAEYVVESTGVFTTMEK/A	2	1147.119	-0.099	9.97	4.92E-20	86-106	0	:::::::::::::::::::Oxidation_M:::
							R/VPTANVSVVDLTCR/L	3	511.014	-0.078	8.55	6.63E-14	234-247	0	:::::::::::::Cys_CAM::
							K/WGDAGAEYVVESTGVFTTMEK/A	2	1139.499	-0.476	8.05	1.97E-12	86-106	0	::::::::::::::::::::::
							R/GALQNIIPASTGAAK/A	2	706.574	-0.175	7.45	3.85E-10	200-214	0	::::::::::::::::
							K/AGAHLQGGAK/R	2	455.279	-0.03	7.33	1.20E-09	107-116	0	:::::::::::
							K/WGDAGAEYVVESTGVFTTMEK/A	3	765.249	-0.233	7.07	5.76E-09	86-106	0	:::::::::::::::::::Oxidation_M:::
							K/WGDAGAEYVVESTGVFTTMEK/A	2	1139.499	-0.476	6.6	9.94E-08	86-106	0	::::::::::::::::::::::
spot 479	Annexin A2	ANXA2	P07355	11	33	81.5	K/SLYYYIQQDTK/G	2	711.374	-0.023	9.82	6.60E-19	314-324	0	::::::::::::
							K/TDLEKDIISDTSGDFR/K	2	906.669	-0.232	9.55	6.77E-18	153-168	1	:::::::::::::::::
							K/TDLEKDIISDTSGDFR/K	3	604.739	-0.112	9.53	1.22E-17	153-168	1	:::::::::::::::::
							K/TPAQYDASELK/A	2	611.964	-0.163	9.38	5.52E-17	105-115	0	::::::::::::
							R/AEDGSVIDYELIDQDAR/D	2	955.194	-0.249	8.93	2.32E-15	180-196	0	::::::::::::::::::
							R/RAEDGSVIDYELIDQDAR/D	3	689.349	-0.35	7.7	1.00E-10	179-196	1	:::::::::::::::::::
							R/TNQELQEINR/V	2	623.124	-0.309	7.47	6.20E-10	136-145	0	:::::::::::
							R/DALNIETAIK/T	2	545.289	-0.985	7.31	2.54E-09	38-47	0	:::::::::::
							R/QDIAFAYQR/R	2	556.439	-0.159	7.29	2.69E-09	69-77	0	::::::::::
							K/WISIMTER/S	2	526.424	-0.158	6.47	8.20E-07	213-220	0	:::::Oxidation_M::::
							K/LSLEGDHSTPPSAYGSVK/A	3	616.084	-0.445	6.28	2.71E-06	11-28	0	:::::::::::::::::::
spot 680	Transgelin-2	TAGLN2	P37802	3	19	25.1	R/DDGLFSGDPNWFPK/K	2	798.224	-0.361	9.74	7.32E-19	139-152	0	:::::::::::::::
							R/NFSDNQLQEGK/N	2	640.524	-0.224	7.32	1.09E-09	160-170	0	::::::::::::
							R/TLMNLGGLAVAR/D	2	616.274	0.071	6.92	2.09E-08	127-138	0	:::Oxidation_M::::::::::
spot 744	Fatty acid-binding protein	FABP4	P15090	39	73	145.9	K/PNMIISVNGDVITIK/S	2	807.839	-0.389	11.7	2.90E-28	38-52	half	::::::::::::::::
							S/FILGQEFDEVTADDR/K	2	878.224	-0.309	11.1	4.38E-25	64-78	half	::::::::::::::::
							K/NTEISFILGQEFDEVTADDR/K	3	767.309	-0.28	11.1	3.21E-25	59-78	0	:::::::::::::::::::::
							K/NTEISFILGQEFDEVTADDR/K	2	1150.059	-0.019	10.7	2.67E-23	59-78	0	:::::::::::::::::::::
							V/SSENFDDYMKEVGVGFATR/K	2	1076.734	-0.248	10.2	5.05E-21	12-30	half	::::::::::::::::::::
							K/PNMIISVNGDVITIK/S	2	815.639	-0.191	10.1	3.12E-20	38-52	half	:::Oxidation_M:::::::::::::
							K/NTEISFILGQEFDEVTADDR/K	3	767.314	-0.285	10	3.68E-20	59-78	0	:::::::::::::::::::::

Additional file 3
Protein identification by Phenyx

							K/NTEISFILGQEFDEVTADDR/K	3	767.064	-0.035	9.6	2.96E-18	59-78	0	:::::::::::::::::::::
							K/STITLDGGVLVHVQK/W	2	784.074	-0.128	9.48	8.96E-18	82-96	0	::::::::::::::::
							K/NTEISFILGQEFDEVTADDRK/V	3	810.049	-0.322	9.27	6.51E-17	59-79	1	::::::::::::::::::::::
							K/LVSSENFDDYMKEVGVGFATR/K	2	1182.409	0.154	9.2	4.14E-17	10-30	1	::::::::::::::::::::::
							F/ILGQEFDEVTADDR/K	2	804.519	-0.138	8.94	1.34E-15	65-78	half	:::::::::::::::
							K/NTEISFILGQEFDEVTADDR/K	2	1150.474	-0.434	8.77	3.72E-15	59-78	0	:::::::::::::::::::::
							K/NTEISFILGQEFDEVTADDRK/V	2	1214.494	-0.406	7.98	1.62E-12	59-79	1	::::::::::::::::::::::
							V/SSENFDDYMKEVGVGFATR/K	2	1084.844	-0.36	7.89	7.65E-12	12-30	half	:::::::::Oxidation_M:::::::::::
							N/MIISVNGDVITIK/S	2	702.299	-0.396	7.77	3.14E-11	40-52	half	::::::::::::::
							K/LVSSENFDDYMK/E	2	724.489	-0.165	7.65	8.04E-11	10-21	0	:::::::::::::
							K/LVSSENFDDYMK/E	2	724.894	-0.57	7.5	2.55E-10	10-21	0	:::::::::::::
							K/REDDKLVVECVMK/G	3	546.729	-0.456	7.33	1.17E-09	108-120	2	::::::::::Cys_CAM::Oxidation_M::
							K/REDDKLVVECVMK/G	3	541.094	-0.153	6.97	1.46E-08	108-120	2	::::::::::Cys_CAM::::
							K/LVVECVMK/G	2	489.389	-0.127	6.93	2.26E-08	113-120	0	:::::Cys_CAM::::
							K/LVSSENFDDYMKEVGVGF/A	2	1026.744	-0.277	6.86	1.88E-08	10-27	half	:::::::::::Oxidation_M::::::::
							K/VAGMAKPNMIISVNGDVITIK/S	3	724.929	-0.527	6.81	3.87E-08	32-52	0	::::::::::::::::::::::
							K/LVSSENFDDYMKEVGVGFATR/K	3	789.194	-0.483	6.8	3.75E-08	10-30	1	::::::::::::::::::::::
							K/STITLDGGVLVHVQKWDGK/S	2	1027.249	-0.191	6.56	1.48E-07	82-100	1	::::::::::::::::::::
							K/LVVECVMK/G	2	497.344	-0.085	6.49	4.24E-07	113-120	0	:::::Cys_CAM::Oxidation_M::
							K/EVGVGFATR/K	2	468.499	-0.248	6.37	1.02E-06	22-30	0	::::::::::
							K/LVSSENFDDYMKEVGVGFATR/K	3	794.369	-0.327	6.15	2.81E-06	10-30	1	:::::::::::Oxidation_M:::::::::::
							K/LVVECVMK/G	2	489.339	-0.077	5.94	1.47E-05	113-120	0	:::::Cys_CAM::::
							K/PNMIISVNGDVITIK/S	2	808.419	-0.969	5.91	1.16E-05	38-52	half	::::::::::::::::
							K/VAGMAKPNMIISVNGDVITIK/S	3	730.114	-0.38	5.88	1.55E-05	32-52	0	::::Oxidation_M::::::::::::::::::
							K/LVSSENFDDYMK/E	2	732.494	-0.172	5.81	2.39E-05	10-21	0	:::::::::::Oxidation_M::
							V/SSENFDDYMK/E	1	1251.35	0.133	5.51	3.84E-05	12-21	half	:::::::::Oxidation_M::
							K/VAGMAKPNMIISVNGDVITIK/S	3	735.364	-0.298	5.5	0.000149	32-52	0	::::Oxidation_M:::::Oxidation_M:::::::::::::
							K/VAGMAKPNMIISVNGDVITIK/S	2	1102.679	-0.584	5.43	0.000141	32-52	0	::::Oxidation_M:::::Oxidation_M:::::::::::::
							F/ILGQEFDEVTADDR/K	3	536.759	-0.169	5.35	0.000456	65-78	half	:::::::::::::::
							K/NTEISFILGQEFDEVTADDR/K	2	1150.224	-0.184	5.3	0.000249	59-78	0	:::::::::::::::::::::
							N/MIISVNGDVITIK/S	2	710.814	-0.914	5.28	0.000493	40-52	half	:Oxidation_M:::::::::::::
							K/STITLDGGVLVHVQK/W	3	523.244	-0.277	5.23	0.000902	82-96	0	::::::::::::::::
spot 801	Lamin-A/C	LMNA	P02545	35	41	197.6	R/NSNLVGAAHEELQQSR/I	2	877.234	-0.299	11.8	9.30E-29	281-296	0	:::::::::::::::::
							K/AAYEAELGDAR/K	2	583.214	0.064	11.3	6.08E-26	79-89	0	::::::::::::
							R/TALINSTGEEVAMR/K	2	754.599	-0.224	11	2.01E-24	528-541	0	:::::::::::::Oxidation_M::
							K/AQNTWGCGNSLR/T	2	682.519	-0.207	9.57	4.61E-18	516-527	0	:::::::Cys_CAM::::::
							R/SGAQASSTPLSPTR/I	2	680.594	-0.247	9.49	9.58E-18	12-25	0	:::::::::::::::
							K/LRDLEDSLAR/E	2	594.439	-0.116	9.42	2.16E-17	320-329	1	:::::::::::
							R/SVGGSGGGSFGDNLVTR/S	2	784.144	-0.265	9.41	1.88E-17	628-644	0	::::::::::::::::::
							R/LKDLEALLNSK/E	2	622.419	-0.052	9.39	2.68E-17	134-144	1	::::::::::::
							R/NSNLVGAAHEELQQSR/I	3	585.379	-0.42	8.95	1.57E-15	281-296	0	:::::::::::::::::
							R/MQQQLDEYQELLDIK/L	2	947.934	-0.467	8.9	1.61E-15	352-366	0	::::::::::::::::
							R/MQQQLDEYQELLDIK/L	3	637.444	-0.132	8.7	1.49E-14	352-366	0	:Oxidation_M:::::::::::::::
							R/SLETENAGLR/L	2	545.369	-0.088	8.68	1.95E-14	51-60	0	:::::::::::
							R/ITESEEVVSR/E	2	574.809	-0.015	8.53	6.63E-14	63-72	0	:::::::::::
							R/IDSLSAQLSQLQK/Q	2	716.459	-0.563	8.47	9.52E-14	299-311	0	::::::::::::::
							R/MQQQLDEYQELLDIK/L	2	955.944	-0.48	8	3.64E-12	352-366	0	:Oxidation_M:::::::::::::::
							R/LRITESEEVVSR/E	3	473.344	-0.084	7.95	1.03E-11	61-72	1	:::::::::::::
							R/TALINSTGEEVAMR/K	2	746.614	-0.237	7.94	7.74E-12	528-541	0	:::::::::::::::
							K/EAALSTALSEK/R	2	560.319	-0.021	7.76	4.44E-11	145-155	0	::::::::::::
							R/VAVEEVDEEGKFVR/L	2	803.829	-0.419	7.55	1.55E-10	440-453	1	:::::::::::::::
							R/LQEKEDLQELNDR/L	3	544.249	-0.308	7.1	6.50E-09	29-41	1	::::::::::::::
							R/VAVEEVDEEGKFVR/L	3	536.079	-0.137	7.04	9.82E-09	440-453	1	:::::::::::::::
							R/MQQQLDEYQELLDIK/L	3	632.194	-0.214	7.02	9.71E-09	352-366	0	::::::::::::::::
							K/LALDMEIHAYR/K	3	450.519	-0.623	6.99	1.55E-08	367-377	0	:::::Oxidation_M:::::::
							R/TLEGELHDLR/G	2	592.379	-0.569	6.92	2.01E-08	157-166	0	:::::::::::
							R/LRITESEEVVSR/E	2	709.559	-0.173	6.84	3.27E-08	61-72	1	:::::::::::::
							K/EGDLIAAQAR/L	2	522.264	0.014	6.72	9.60E-08	124-133	0	:::::::::::
							R/TALINSTGEEVAMR/K	3	503.654	-0.402	6.7	1.09E-07	528-541	0	:::::::::::::Oxidation_M::
							R/LQTMKEELDFQK/N	3	509.564	-0.307	6.26	2.10E-06	197-208	1	::::Oxidation_M:::::::::
							R/LQTMKEELDFQK/N	3	504.224	-0.299	6.23	2.41E-06	197-208	1	:::::::::::::
							K/NIYSEELR/E	2	512.339	-0.08	5.96	1.30E-05	209-216	0	:::::::::
							R/LQTMKEELDFQK/N	2	763.539	-0.157	5.96	9.67E-06	197-208	1	::::Oxidation_M:::::::::
							R/LSPSPTSQR/S	2	486.824	-0.065	5.5	0.000207	389-397	0	::::::::::
							K/LEAALGEAK/K	2	451.154	0.099	5.44	0.000302	172-180	0	::::::::::
							R/LADALQELR/A	2	514.954	-0.163	5.42	0.000312	241-249	0	::::::::::
							R/SGAQASSTPLSPTR/I	2	682.004	-1.657	5.18	0.00094	12-25	0	:::::::::::::::
spot 841	94 kDa glucose-regulated protein	GRP94	P14625	15	18	105.2	R/EEEAIQLDGLNASQIR/E	2	893.564	-0.111	13.1	1.46E-35	31-46	0	:::::::::::::::::
							R/LISLTDENALSGNEELTVK/I	2	1023.809	-0.275	10.4	7.32E-22	96-114	0	::::::::::::::::::::
							R/ELISNASDALDK/I	2	638.379	-0.054	10	4.90E-20	82-93	0	:::::::::::::
							R/EEEAIQLDGLNASQIR/E	3	596.024	-0.053	9.42	2.19E-17	31-46	0	:::::::::::::::::
							K/DISTNYYASQK/K	2	645.464	-0.16	9.02	8.40E-16	651-661	0	::::::::::::
							K/GVVDSDDLPLNVSR/E	2	743.489	-0.108	8.88	2.54E-15	414-427	0	:::::::::::::::
							R/LISLTDENALSGNEELTVK/I	3	682.739	-0.047	8.87	3.00E-15	96-114	0	::::::::::::::::::::
							K/LGVIEDHSNR/T	2	570.569	-0.275	8.86	3.52E-15	473-482	0	:::::::::::

Additional file 3

Protein identification by Phenyx

							R/TDDEVVQREEEAIQLDGLNASQIR/E	3	910.659	-0.541	8.51	5.52E-14	23-46	1	:::::::::::::::::::::::::
							R/GLFDEYGSK/K	2	508.419	-0.179	8.43	1.90E-13	375-383	0	::::::::::
							R/LISLTDENALSGNEELTVK/I	2	1025.404	-1.87	8.08	1.74E-12	96-114	0	::::::::::::::::::::
							K/NLLHVTDTGVGMTR/E	3	510.284	0.312	7.63	1.26E-10	122-135	0	::::::::::::Oxidation_M:::
							R/LSLNIDPDAK/V	2	543.349	-0.053	6.97	1.65E-08	724-733	0	:::::::::::
							K/FAFQAEVNR/M	2	541.434	-0.159	6.96	1.57E-08	55-63	0	::::::::::
							K/SGTSEFLNK/M	2	491.804	-0.058	6.16	3.68E-06	148-156	0	::::::::::
spot 906	NADH-ubiquinone oxidoreductase 30 kDa subunit	NDUFS3	O75489	10	46	84.3	K/QLSAFGEYVAEILPK/Y	2	833.249	-0.3	12.4	1.06E-31	21-35	0	::::::::::::::::
							K/DFPLSGYVELR/Y	2	648.904	-0.569	10.9	6.38E-24	165-175	0	::::::::::::
							K/SLVDLTAVDVPTR/Q	2	693.554	-0.168	10.5	3.50E-22	74-86	0	::::::::::::::
							R/VVAEPVELAQEFR/K	3	496.379	-0.111	9.55	6.72E-18	183-195	0	::::::::::::::
							K/TYTDELTPIESAVSVFK/A	2	950.739	-0.256	8.91	1.44E-15	109-125	0	::::::::::::::::::
							R/VVAEPVELAQEFR/K	2	744.154	-0.255	8.67	1.59E-14	183-195	0	::::::::::::::
							K/RVVAEPVELAQEFR/K	3	548.389	-0.087	8.58	4.51E-14	182-195	1	:::::::::::::::
							R/KFDLNSPWEAFPVYR/Q	3	623.794	-0.145	8.4	2.12E-13	196-210	1	::::::::::::::::
							R/ILTDYGFEGHPFR/K	3	518.129	-0.204	8.22	1.03E-12	151-163	0	::::::::::::::
							K/AANWYER/E	2	455.314	-0.099	6.83	4.27E-08	126-132	0	::::::::
spot 912	Triosephosphate isomerase	TPI	P60174	12	42	77.1	K/SNVSDAVAQSTR/I	2	617.904	-0.099	12.6	6.71E-33	194-205	0	:::::::::::::
							R/HVFGESDELIGQK/V	2	730.189	-0.324	11.1	6.54E-25	100-112	0	::::::::::::::
							R/IIYGGSVTGATCK/E	2	664.264	-0.424	9.76	7.33E-19	206-218	0	::::::::::::Cys_CAM::
							K/DCGATWVVLGHSER/R	2	793.939	-0.066	8.76	6.91E-15	85-98	0	::Cys_CAM:::::::::::::
							K/DCGATWVVLGHSER/R	3	529.879	-0.295	8.71	1.63E-14	85-98	0	::Cys_CAM:::::::::::::
							R/HVFGESDELIGQK/V	3	487.044	-0.132	8.1	3.15E-12	100-112	0	::::::::::::::
							R/RHVFGESDELIGQK/V	3	539.084	-0.138	7.92	1.23E-11	99-112	1	:::::::::::::::
							K/VTNGAFTGEISPGMIK/D	2	820.104	-0.69	7.86	1.33E-11	69-84	0	::::::::::::::Oxidation_M:::
							K/VIADNVKDWSK/V	2	637.994	-0.153	6.85	3.30E-08	149-159	1	::::::::::::
							K/IAVAAQNCYK/V	2	569.464	-0.174	6.43	6.19E-07	59-68	0	::::::::Cys_CAM:::
							K/TATPQQAQEVHEK/L	3	489.919	-0.34	5.65	8.29E-05	175-187	0	::::::::::::::
							K/VTNGAFTGEISPGMIK/D	2	812.299	-0.882	5.62	6.61E-05	69-84	0	:::::::::::::::::
